# Neck Chamber Technique Revisited: Low-Noise Device Delivering Negative and Positive Pressure and Enabling Concomitant Carotid Artery Imaging With Ultrasonography

**DOI:** 10.3389/fphys.2021.703692

**Published:** 2021-10-05

**Authors:** Rafał Seredyński, Tymoteusz Okupnik, Przemysław Musz, Stanisław Tubek, Beata Ponikowska, Bartłomiej Paleczny

**Affiliations:** ^1^Department of Physiology and Pathophysiology, Wrocław Medical University, Wrocław, Poland; ^2^IMER Systems, Wrocław, Poland; ^3^Institute of Heart Diseases, Wroclaw Medical University, Wrocław, Poland

**Keywords:** neck chamber method, baroreflex sensitivity, carotid artery ultrasonography, neck suction, carotid baroreceptor

## Abstract

**Background and Objectives:** Recently, novel noiseless device for the assessment of baroreceptor function with the neck suction (NS) has been presented. In this study, we present another in-house approach to the variable-pressure neck chamber method. Our device offers further critical improvements. First, it enables delivery of negative (NS) as well as positive pressure (neck pressurizing, NP) in a noiseless manner. Second, we used small, 3D-printed cups positioned over the carotid sinuses instead of cumbersome neck collar to improve subject comfort and to test feasibility of tracking the pressure-induced changes in carotid artery with ultrasonography.

**Methods:** Five healthy, non-smoking, normal-weight subjects aged 29 ± 3 years (mean ± SD) volunteered for the study. Heart rate (HR, bpm) and mean arterial pressure (MAP, mmHg) responses to short, 7-s long episodes of NS and NP were recorded. Each trial consisted of 12 episodes of variable-pressure: six episodes of NS (suction ranging between -10 and -80 mmHg) and six episodes of NP (pressure ranging between + 10 and + 80 mmHg). Carotid artery sonography was performed during the NS and NP in four subjects, on another occasion.

**Results:** The variable-pressure episodes resulted consistently in the expected pattern of hemodynamic alterations: HR and MAP increases or decreases following the NP and NS, respectively, as evidenced by the coefficient of determination (R^2^) of ≥0.78 for the carotid-HR response curve (for all five participants) and the carotid-MAP response curve (for four out of five participants; the curve cannot be calculated for one subject). We found a linear, dose-dependent relation between the applied pressure and the systolic-diastolic difference in carotid artery diameter.

**Conclusion:** The novel device enables noiseless stimulation and unloading of the carotid baroreceptors with the negative and positive pressure, respectively, applied on the subject’s neck via small, asymmetric and one-side flattened, 3D-printed cups. The unique design of the cups enables concomitant visualizing of the carotid artery during the NS or NP administration, and thereby direct monitoring of the intensity of mechanical stimulus targeting the carotid baroreceptors.

## Introduction

The past decade has witnessed the advent of several novel therapies designed for certain cardiovascular disorders (i.e., heart failure and resistant hypertension) that target the autonomic nervous system, including the carotid baroreceptor reflex (Victor, 2015; Spiering et al., 2017; van Bilsen et al., 2017). This has been accompanied by a growing body of research on physiology and methods for testing the carotid baroreceptor function (De Leeuw et al., 2015; Pinna et al., 2015, 2017; Pinheiro et al., 2020). The current paper fits into this trend by advancing the technical aspects and the understanding of the physiological effects of the neck chamber (collar) method.

The arterial baroreceptors are stretch-sensitive afferents embedded in the walls of the large vessels (the carotid sinus and the aortic arch, mainly). They detect deformation of the vessel wall resulting from the changes in the mean arterial pressure, primarily, while the pulse pressure is believed to provide additional contribution to baroreceptor activity (Brown, 1980; Koushanpour, 1991; Chapleau et al., 2001). A rise in the blood pressure increases the neural input from the baroreceptors to brainstem autonomic centers and leads to peripheral vasodilation (via suppression of tonic vasoconstrictor outflow to the vasculature) and decreased heart rate (due to increased vagal and reduced sympathetic activity to the heart). Consequently, the blood pressure is maintained within the normal range. Conversely, rapid fall in arterial blood pressure results in baroreceptor unloading due to less stretch of the arterial wall, leading to peripheral vasoconstriction and increased heart rate, both contributing to blood pressure recovery (Koushanpour, 1991; Parati et al., 2000; Fadel et al., 2003; La Rovere et al., 2008). Therefore, under physiological conditions, the arterial baroreflex acts to buffer short-term wide fluctuations of the arterial blood pressure rather than to set the chronic level of arterial blood pressure (Cowley et al., 1973). In contrast, however, observations from clinical trials suggest that chronic lowering of blood pressure in hypertensive patients is achievable with sustained electrical (Hoppe et al., 2012; Abraham et al., 2015; Victor, 2015; van Bilsen et al., 2017) or mechanical (Spiering et al., 2017) stimulation of carotid baroreceptors.

Various experimental approaches for testing the baroreceptor mechanism have been developed (Parati et al., 2000; La Rovere et al., 2008). In brief, the baroreceptor function can be quantified as: (i) the cardiac or sympathetic response to blood pressure perturbations produced by iv injection of vasoconstrictors (phenylephrine), vasodilators (sodium nitroprusside), or both in sequence (the modified Oxford method) (Smyth et al., 1969; La Rovere et al., 2008); (ii) the cardiac or sympathetic response to spontaneous, beat-to-beat, oscillations of blood pressure (i.e., the sequence method) (Parati et al., 1988; La Rovere et al., 2008); (iii) the cardiac, vascular, blood pressure or sympathetic response to mechanical stimulation of the carotid baroreceptors with negative or positive pressure applied on the subject’s neck, over the carotid sinuses (Ludbrook et al., 1977; Fadel et al., 2003; Cooper and Hainsworth, 2009; Pinheiro et al., 2020). The last of the listed, known as the variable-pressure neck chamber (collar) method, carries several advantages over the other techniques. The method is non-invasive; allows for precise control over the stimulus (its duration and intensity) and can be used under various conditions (i.e., exercise). Furthermore, unlike the other methods, the neck chamber technique enables the vascular and pressure components of the arterial baroreflex to be determined (Ludbrook et al., 1977; Fadel et al., 2003; Cooper and Hainsworth, 2009). Finally, while the other methods explore the middle linear portion of the baroreflex stimulus-response curve only, the neck chamber technique can be used to identify the threshold and saturation points of the curve as well. Consequently, the entire sigmoidal curve of the baroreceptor response can be modeled, thereby allowing for a comprehensive assessment of the operational parameters of the carotid baroreflex, including the operating range and responding range (Kent et al., 1972; Fadel et al., 2003). Despite all the mentioned advantages that make the neck chamber technique an invaluable tool for studying the baroreceptor physiology, it has been rarely used in the clinical setting, possibly due to the lack of commercially available and widely accepted equipment and little reference data available (Fadel et al., 2003; Cooper and Hainsworth, 2009).

Recently, Pinheiro et al. (2020) have revisited the neck chamber method and demonstrated a novel low-noise device. Indeed, loud noise generated by custom-built systems substantially limits the applicability of the method in clinical practice (Cooper and Hainsworth, 2009; Pinheiro et al., 2020) and is a possible confounder that can impact the autonomic control of cardiovascular function (Lee et al., 2010; Pinheiro et al., 2020).

Encouraged by the report of Pinheiro et al. (2020), we present an alternative approach for the assessment of baroreflex sensitivity with the neck chamber method. The equipment utilized in our center, while being as low-noise as that of Pinheiro et al. (2020), offers further improvements: (i) negative or positive pressure can be delivered, thus allowing to reconstruct the entire sigmoidal curve of the baroreceptor response; (ii) a pair of small, 3D-printed cups positioned bilaterally on the subject’s neck, over the carotid sinuses, is used instead of heavy and cumbersome neck collar to improve subject comfort; and (iii) the cups are asymmetric and flattened on one side to allow for visualizing the carotid artery with ultrasonography [ultrasound, United States (US)] *during* the test.

In the current paper, we aimed to test the feasibility and safety of the carotid baroreceptor stimulation and unloading with our device for the neck-chamber method. We assessed the performance of our device in terms of the level of noise generated during its operation, the effectiveness of carotid baroreceptor stimulation/unloading, and the feasibility of visualizing the carotid artery with US *during* the test.

## Materials and Methods

### Ethical Approval and Study Population

The study protocol was approved by the local Institutional Ethics Committee (Bioethics Committee of the Wrocław Medical University). Each participant gave his or her informed written consent. The study conformed to the standards established by the *Declaration of Helsinki*. The study involved five healthy, non-smoking, recreationally active, normal-weight volunteers (three males), aged between 26 and 34 years (mean ± SD: 29 ± 3 years). Before the examination, subjects abstained from caffeine and physical exercise for a minimum of 24 h.

### Neck Chamber Device

#### Negative- and Positive Pressure Generators

**FIGURE 1:**
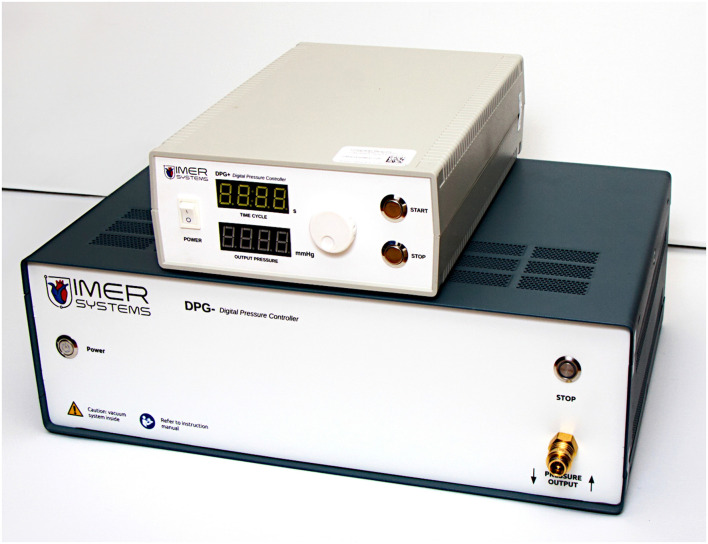
The neck chamber device used in the study.

**FIGURE 2:**
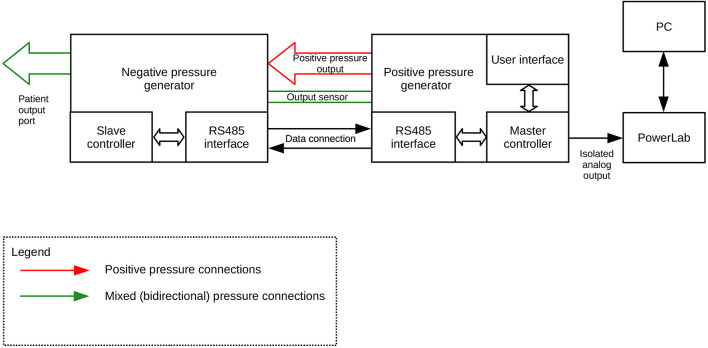
Diagram of the connections between the negative- (DPG-) and positive pressure (DPG+) generator.

**FIGURE 3:**
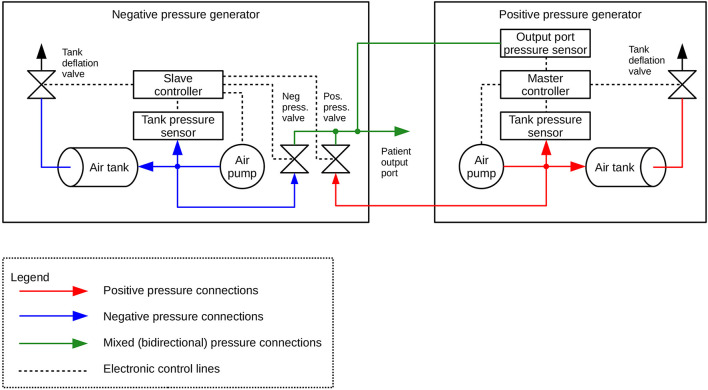
Diagram of the construction of the negative- (DPG-) and positive pressure (DPG+) generator.

We used a custom-designed system consisting of two pressure generation units working in a master-slave mode (Figures 1–3). The positive pressure generator (called DPG +) acts as a master for controlling the negative pressure (DPG-) slave unit. The DPG + device is equipped with an embedded user interface, consisting of an encoder wheel (for setting time and pressure level and also for calling autocalibration function), two control switches (START and STOP, the latter can be used as emergency stop button), and two 7-segment, 4-position LED displays for displaying pressure level, time and system alerts during calibration, autotest and also error states. For safety purposes, the DPG- unit also has its own STOP button, logically connected (through the Rs485 data interface) with the appropriate button of the DPG + device.

Both units are interconnected using one 4-wire cable (for power supply and data exchange through RS485 differential bus, working in half-duplex mode), one elastic pipe (for working pressure, 6 mm internal diameter, 11 mm external diameter), and one rigid pipe (for output sensor probing line, 2 mm internal diameter, 4 mm external diameter). Depending on which range of pressure is set by the user, the system uses a proper pneumatic subsystem (positive or negative pressure generator). During the procedure, the unused subsystem is turned off and being cut off by its output valve. Both output valves are connected, forming the subject output pressure port. This is also the point of probing the output pressure by the main pressure sensor, embedded in the DPG + master device and connected to the air mixer by the 4 mm pipe mentioned above.

Every unit has ca. 1-liter air tank equipped with voltage-controlled deflation valve and low-noise, low-power pumping system consisting of one or two membrane pumps. The pressure setpoint for each tank is automatically derived from autocalibration data that should be done by the user after every change in the output pressure setting. This feature allows precise pumping of the tanks and thus compensation of dynamic pressure differences caused by elastic susceptibility of the suction/pressure cups.

Both devices are based on 32-bit ARM Cortex-M0 microcontrollers (STM32F0 family, ST Microelectronics). 6V DC and 12V DC, direct acting control valves are used because of rapid response, low power and low voltage supply, small size and quiet operation. The valves are controlled by integrated power switches utilizing hardware over-current and thermal protection. For pressure/vacuum sensing we use a high-precision digital pressure sensors (± 15 psi and 1% accuracy for vacuum tank, 0-5 psi and 0.25% accuracy for positive pressure tank and ± 1 psi and 0.25% accuracy for subject output port monitoring). We also designed a ESD-protected RS485 differential data bus for communication between both microcontrollers, using a custom-developed binary protocol with data integrity ensured by CRC check. Subject port pressure can be read by an external DAQ via an single-ended analog output provided by a built-in 12-bit D/A converter and an isolation amplifier, that acts as a reinforced isolation providing high level of subject safety. The system is powered by a medical-grade AC power adaptor (Mean Well), classified as a suitable for BF application part (2 MOPP).

The neck chamber device can be considered a portable device, given its physical dimensions of 280 mm × 200 mm × 85 mm for DPG + and 435 mm × 280 mm × 135 mm for DPG-.

#### Neck Chambers

**FIGURE 4:**
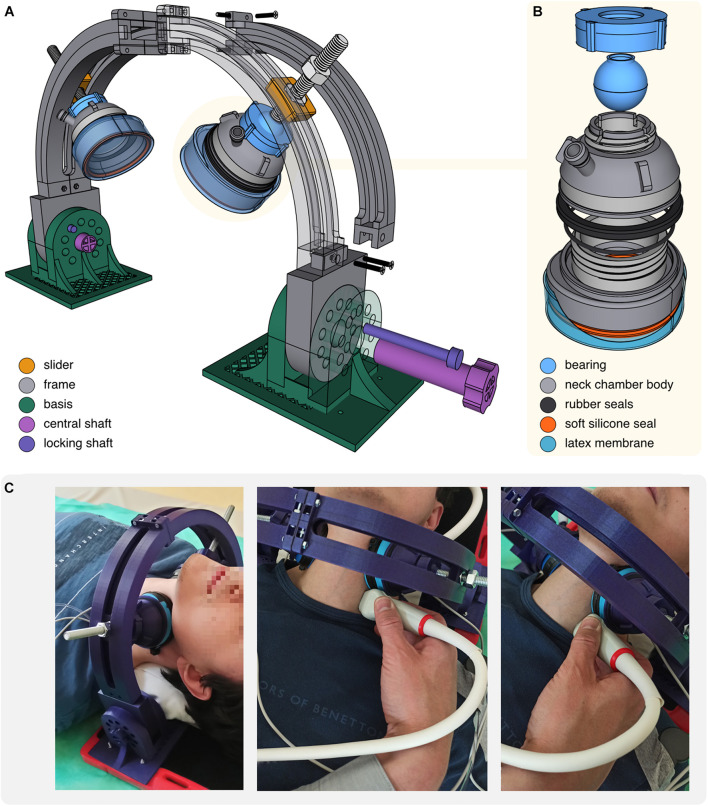
**(A)** Scheme of the neck chambers (cups) mounted on the frame. **(B)** Scheme of the neck chamber (cup). **(C)** Photographs of the neck chambers positioned on the subject’s neck. Positioning of the US probe during the US examination of the carotid artery was shown.

The neck chamber set consists of two cups mounted on an adjustable frame (Figure 4A). All major parts were modeled with Shapr3D software (Shapr3D Zrt., Hungary, Budapest) and printed with Prusa i3 MK3s 3D printer (Prusa Research, Prague, Czech Republic) using polylactide (PLA) filament.

The major challenge with the neck chambers design was the adjustment of their shape and size to obtain: (i) firm adherence to the neck surface under both positive and negative pressures, (ii) neck coverage sufficient to elicit baroreflex response, and (iii) compactness, to provide space for the US probe. To achieve these objectives, we modeled oval, asymmetric cups (71 mm × 67 mm, the inner surface area of 28 cm^2^), flattened on the side directed toward the US probe (Figures 4B,C). Applying some principles described by Raine and Cable (1999), we closed the bottom of each cup with a very thin, skin-adhesive latex membrane. Under negative pressure (NS, NS) membrane indents inside the cup, pulling up and spreading the underlying tissues; under positive pressure (neck pressure, NP) inflated membrane distends against the skin and produces compression. Of note, neck chamber components can be easily disassembled, allowing quick membrane replacement after each use (Figure 4B). To ensure tightness, segments are sealed with rubber and silicone rings (available in standard plumbing repair kits).

The neck chamber set was intended to enable simultaneous ultrasonographic measurements, whereas cup movements (especially during inflation) might severely distort the US recordings. Therefore, we decided to mount chambers on the rigid frame (Figure 4A) providing four adjustment levels to ensure firm and stable cup placement on the subject’s neck: (i) bearings allow to freely rotate and tilt the cup, as well as lock it in the desired position; (ii) M10 screws allow to press the cup against the neck in a vice-like manner; (iii) M10 screws are seated in sliders moving along the frame; (iv) frame can be tilted forward and backward.

3D models of all printable parts are provided in the supplement as (non-editable).stl files. Editable version (.obj or .step) is available from the corresponding author upon reasonable request.

We have identified two drawbacks of the novel device, both being probably the result of relatively low air volume enclosed by the cups. First, typically there is a progressive loss of the pressure generated beneath the cups over time. The mean difference between the pressure at the onset of the pressure application and the pressure at the 5^*th*^ second of the pressure application was: −6.70 ± 1.95 mmHg for the NS of −20 mmHg, −12.84 ± 4.36 mmHg for the NS of −60 mmHg, 3.83 ± 1.45 mmHg for the NP of + 20 mmHg, and 7.25 ± 3.46 mmHg for the NP of +60 mmHg (see Supplementary File for further detail). This is likely attributable to a change in air volume enclosed by the cups resulting from neck tissue expanding or compressing under negative and positive pressure, respectively, since virtually no pressure loss was observed when the cups were used on the non-deformable surfaces like flat glass or wooden block. The standard neck chamber (collar) systems are characterized by an excellent capability of maintaining stable pressure due to the following reasons: (i) the air volume enclosed by the collar is relatively high, and therefore the pressure-induced expansion/compression of the neck tissue does not affect it significantly, (ii) these systems operate under continuous (although limited) leakage of the air and were designed to quickly compensate for this leakage.

**FIGURE 5:**
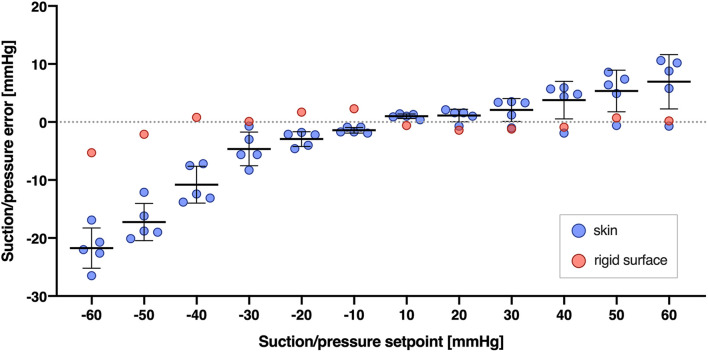
Difference between the NP/NS value programmed and the actual pressure generated beneath the cups (Suction/pressure error, y axis) across different pressures programmed (Suction/pressure setpoint, x axis), and for two types of surface: skin (blue circles) and a rigid, glass surface (red circles). Suction/pressure error was calculated as the actual pressure generated minus the pressure programmed. Bars and whiskers indicate means ± SD and were shown for skin surface (blue circles) only. The pressures were calculated as the average from 5 s of the suction or pressure trial.

Second, typically there is a small difference between the pressure programmed and the pressure generated beneath the cups (see Figure 5 and Supplementary File for further details). It seems to be a proportional bias increasing as the absolute value of the pressure increases (Figure 5). We believe the difference results from the reasons discussed above, given that it becomes negligible when the cups are applied on the non-deformable surface.

#### Measuring Equipment

Heart rate (HR, bpm) was calculated from the ECG signal (BioAmp; ADInstruments, Dunedin, New Zealand). Hemodynamic parameters [systolic blood pressure (SBP, mmHg), diastolic blood pressure (DBP, mmHg), and mean arterial pressure (MAP, mmHg)] were derived from continuous and non-invasive recording of finger arterial pressure with the device employing the volume-clamp technique (Human NIBP System; ADIntruments). The respiratory movements were collected using a respiratory belt placed around the abdomen (ADIntruments). All data were recorded and stored at a sampling frequency of 1 kHz using a data acquisition system (PowerLab 16/30, ADInstruments). Prior to the examination, the carotid sinuses were located by US. A portable US system (MyLab Gamma, Esaote, Italy) and a high-frequency linear probe (Esaote SL3116) were used. All US examinations were performed by the same, certified sonographer (ST) with extensive experience in the carotid artery US examination. To measure the carotid artery diameter during the negative and positive pressure application, US video recordings of the carotid artery examination in B-mode, in a longitudinal section, were collected and analyzed offline with the US module of the VasoTracker software (Lawton et al., 2019).

After ensuring that all the devices are set to recording data, the neck chambers were comfortably positioned on the neck.

#### Study Protocol

After explaining the protocol, written informed consent was obtained from all participants. The study was performed with the subject lying supine, in a quiet, light-attenuated room with a stable ambient temperature. After about 5 min of the familiarization period, a 10-min baseline period including one episode of voluntary end-expiratory breath-hold was recorded. An 8-min period of acceptable quality, not containing the breath-hold period was selected to calculate baseline values of hemodynamic parameters. Then the NS and NP episodes were conducted.

Trials of the NS were performed in the steps of 10 mmHg starting from −10 mmHg to −60 mmHg and, analogously for the NP – in the steps of 10 mmHg starting from + 10 mmHg to +60 mmHg (each test comprised 12 trials). The NS/NP episodes were interspersed with >120 s gaps between the consecutive trials to allow for the recovery of the examined variables to the baseline values. Each variable-pressure episode was performed during 10- to 15-s end-expiratory breath-hold to avoid respiratory-related modulation of HR (Eckberg, 1976). Prior to the NS or pressure, the operator set the value (mmHg) and the duration (seconds) of the stimulus on the controller. The length of the NS/NP employed in the previous studies was 5 s (Ogoh et al., 2002; Pinheiro et al., 2020), 10 s (Ludbrook et al., 1977; Grassi et al., 1999), or 20 s (Raine and Cable, 1999; Ogoh et al., 2003a). It has been hypothesized that the initial 5 s of the NS/NP reflect the unmodified ‘pure’ response of the carotid baroreceptors, which is quickly followed and modified by secondary responses from the other autonomic reflexes (Cooper and Hainsworth, 2009). Therefore, we decided to analyze the initial 5 s of the NS/NP. However, the duration of a single variable-pressure trial was set at 7 s, in order to exclude the potential effect of pressure release from the analysis.

The pre-stimulus HR and MAP values were calculated by averaging data recorded in the range of three cardiac cycles preceding the onset of the variable-pressure episode. The stimulus HR and MAP values were calculated from the lowest values (nadirs) obtained during the initial 5 s of the NS and, analogously, from the highest values (peaks) obtained during the initial 5 s of the NP. The magnitude of HR and MAP responses was calculated as the difference between the pre-stimulus and stimulus values. Individual stimulus-response curves were evaluated by plotting the stimulus HR and MAP values against the estimated carotid sinus pressure (ECSP), calculated as the pre-stimulus MAP minus neck chamber pressure (Fadel et al., 2003). Each stimulus-response curve was then fitted to the four-parameter logistic function model, as described by Kent and colleagues (Kent et al., 1972; Fadel et al., 2003), using the GraphPad Prism 8 software (GraphPad Software, United States). The following variables were calculated: threshold and saturation, points where no further increase or decrease (respectively) in the HR/MAP occurred despite reductions or increases in ECSP; centering point, ECSP required to elicit equal pressor and depressor responses; operating point, ECSP at which the pre-stimulus HR/MAP value is located. Threshold and saturation values were calculated with equations proposed by Chen and Chang (1991).

To test the feasibility of the carotid artery visualizing with US during the NS/NP application, the above described procedure was repeated with the concomitant carotid artery US examination in four subjects, on another occasion. The systolic-diastolic difference in the carotid artery diameter (CAD_*SYS–DIA*_) was used to assess the effect of the variable-pressure on the carotid artery. Carotid artery diameter values were obtained for each cardiac cycle and averaged for 20 cardiac cycles preceding the NS/NP delivery (the pre-stimulus value) and 5 cardiac cycles following the onset of the NS/NP (the stimulus value). Percentage change in the CAD_*SYS–DIA*_ was calculated as: (pre-stimulus value minus stimulus value)/pre-stimulus value × 100. The results of this experiments were not included in the group analysis of hemodynamic responses to the NS/NP.

Data are presented as mean ± standard deviation (SD) or as mean ± standard error of the mean (SEM).

## Results

The baseline values of the examined variables were: HR, 71 ± 10 bpm; SBP, 130 ± 27 mmHg; DBP, 68 ± 22 mmHg, and MAP, 88 ± 24 mmHg. The mean values of examined variables during breath-hold were as follows: HR, 74 ± 8 bpm; SBP, 129 ± 25 mmHg; DBP, 74 ± 18 mmHg, and MAP 92 ± 21 mmHg.

### Level of Noise Produced by the Device

The mean noise generated by the device, calculated from 5 consecutive trials, was 35 ± 2 dB.

### Physiological Responses to the Neck Suction/Pressure

**FIGURE 6:**
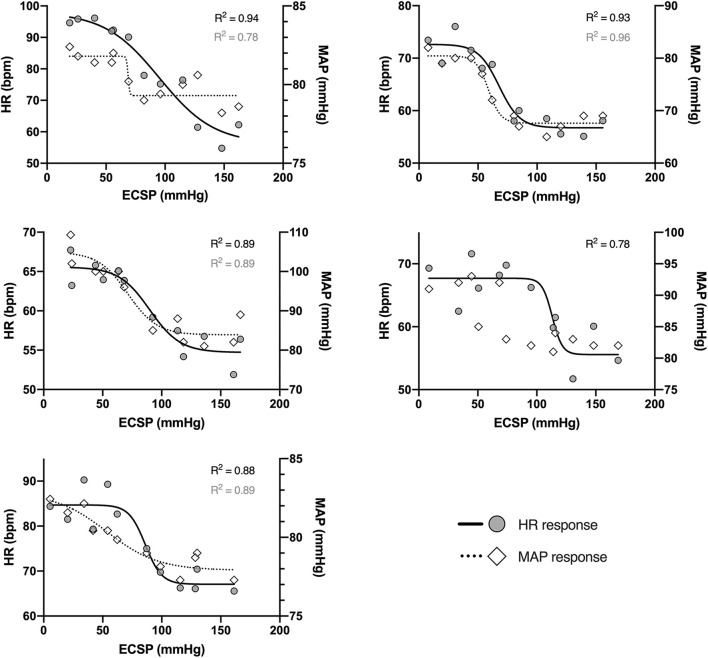
Individual heart rate (filled circles, solid lines; left axis) and mean arterial pressure (empty diamonds, dotted lines; right axis) responses to changes in the estimated carotid sinus pressure (ECSP), collected from five study participants. Estimated carotid sinus pressure was calculated as pre-stimulus MAP minus neck chamber pressure. Each data point represents the peak of the HR/MAP response during each level of neck pressure or suction. Lines represent mean data fitted to logistic function; R^2^ values for solid lines are marked in black, and for dotted lines – in gray.

**FIGURE 7:**
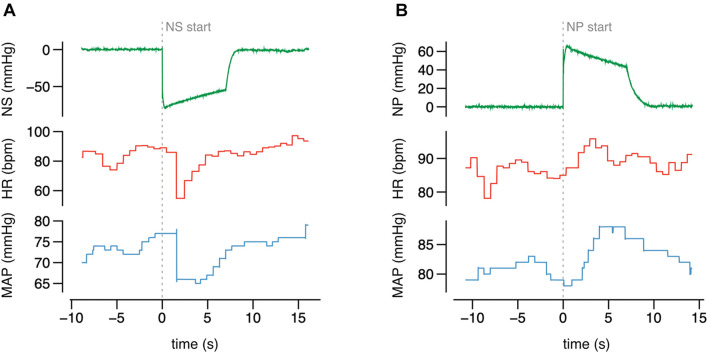
The original recording of HR and MAP during the neck suction (NS) **(A)** and pressure (NP) **(B)** of selected value, collected from one study participant.

Consistent and apparently dose-dependent HR responses to the NS/NP administrated with the novel device were observed in all study participants (Figure 6). Representative tracing collected from one participant was presented in Figure 7. Coefficient of determination (R^2^) calculated for the carotid-HR baroreflex response curve was ≥0.78 in all five participants (Figure 6), demonstrating prominent increase in HR when the ECSP decreased (NP episodes), and decrease in HR when the ECSP increased (NS episodes).

The carotid-MAP response curve cannot be drawn for one participant (Figure 6). For the remaining four subjects, R^2^ for the carotid-MAP response curve was ≥0.78 (Figure 6), and the ECSP falls (NP) and rises (NS) were accompanied by clear MAP increases and decreases, respectively. Detailed characteristics of the stimulus-response relationship for the baroreflex control of HR and MAP are summarized in Table 1.

**TABLE 1:** Variables describing the stimulus-response relationship for the carotid baroreflex control of heart rate (HR) and mean arterial pressure (MAP).

HR responses (*n* = 5)	
Threshold (mmHg)	43.33 ± 14.27
Saturation (mmHg)	134.51 ± 10.16
Response range (bpm)	18.41 ± 4.74
Centering point (mmHg)	90.41 ± 7.30
Operating point (mmHg)	89.86 ± 5.59
HR_*cp*_ (bpm)	68.53 ± 4.26
HR_*op*_ (bpm)	69.54 ± 4.83

**MAP responses (*n* = 4)**	

Threshold (mmHg)	21.80 ± 4.31
Saturation (mmHg)	104.13 ± 15.57
Response range (mmHg)	15.89 ± 2.01
Centering point (mmHg)	63.03 ± 4.14
Operating point (mmHg)	66.01 ± 3.75
MAP_*cp*_ (mmHg)	81.79 ± 3.85
MAP_*op*_ (mmHg)	77.67 ± 4.08

*Values are shown as means ± SEM.*

*HR_*cp*_, heart rate at centering point of reflex; HR_*op*_, heart rate at operating point of reflex; MAP_*cp*_, mean arterial pressure at centering point of reflex; MAP_*op*_, mean arterial pressure at operating point of reflex.*

### Visualizing the Carotid Artery With Ultrasonography

**FIGURE 8:**
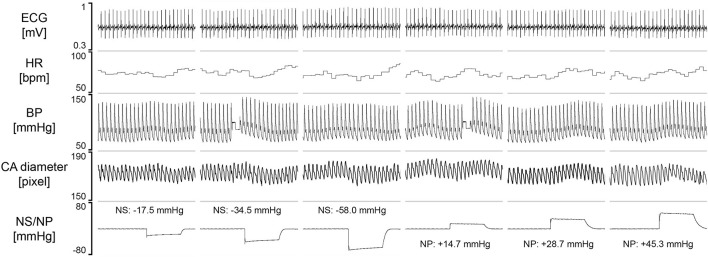
The original recording of the carotid artery diameter with US and physiological variables, collected from one study participant. Values of the neck suction (NS) and neck pressure (NP) are the averages calculated from the initial 5 s of the variable-pressure trial. HR, heart rate; BP, blood pressure; CA, carotid artery.

**FIGURE 9:**
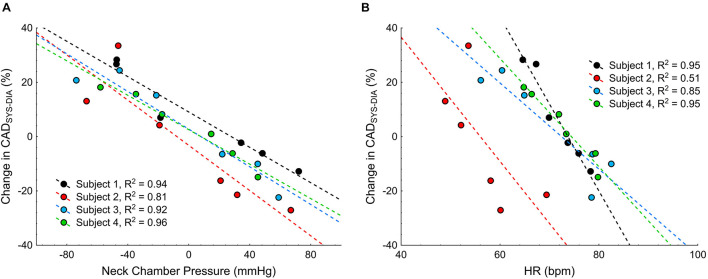
Scatterplots of the change in the systolic-diastolic difference of the carotid artery diameter (CAD_*SYS–DIA*_) vs.: **(A)** the neck chamber pressure, and **(B)** the change in heart rate (HR).

To test the feasibility of visualizing the changes in the carotid artery diameter during the NS or NP, test procedure was repeated with concomitant sonography of the carotid artery in four subjects, on another day. Original tracings of ECG, HR, BP and carotid artery diameter during the NS and NP from one participant are shown in Figure 8. The negative pressures applied on the subject’s neck resulted in clear increases in CAD_*SYS–DIA*_ (Figures 8, 9A). Analogously, the positive pressures resulted in decreases in CAD_*SYS–DIA*_ (Figures 8, 9A). Linear, apparently dose-dependent relations for the change in CAD_*SYS–DIA*_ vs. NS/NP (Figure 9A), and the change in CAD_*SYS–DIA*_ vs. the change in HR (Figure 9B) were found for all the subjects. The video recordings of the US examination performed during the NS of −60 mmHg and the NP of +60 mmHg in one participant, analyzed with the VasoTracker software, have been added as the Supplementary Material.

## Discussion

We believe our device represents the next step in the evolution of the neck chamber method. Founded on the elegant approach described by Pinheiro et al. (2020), the novel device offers further critical improvements, while maintaining the major advantage of Pinheiro’s apparatus – noiseless operation. First, we have demonstrated that the criterion of noiseless operation does not preclude the use of positive pressure for the baroreceptor unloading. In contrast to Pinheiro’s apparatus, the novel device delivers negative and positive pressure (neck suction, NS and neck pressure, NP) and thereby allows for studying the baroreflex mechanism over the entire range of the response, from the threshold to the saturation (Kent et al., 1972). Second, we have replaced the neck collar with small, 3D-printed cups positioned bilaterally on the subject’s neck, over the carotid sinuses. The concept is not new and a handful of studies utilized a similar approach [i.e., neck chambers adapted from ear protectors (Raine and Cable, 1999; Ogoh et al., 2002)]. However, a distinguishing feature of our proposal is asymmetric, one side-flattened cup that allows the high-frequency linear US probe to be placed in close proximity to the pressurized area. Furthermore, since the 3D printing technology has become widely accessible, the neck chamber projects as proposed by us or Pinheiro et al. (2020) can be easily adopted by other centers. Therefore, the files with the printable projects of the cups and frame are available from the corresponding author upon request.

To our knowledge, only one study to date has attempted to visualize the carotid artery during the NS or pressurizing in humans (Kober and Arndt, 1970). Kober and Arndt (1970) used the chamber enclosing the subject’s neck and head. Although the general idea behind the Kober and Arndt’s approach and the contemporarily used neck chambers or neck collars is similar, the results of Kober and Arndt cannot be translated directly to studies utilizing neck chamber- or collar-based systems, given that exposing the entire surface of the head and neck to variable-pressure is likely to trigger multiple local and reflex mechanisms. Therefore, we are the first to demonstrate the feasibility of imaging the carotid artery with US *during* the NS and NP using the contemporary neck chamber-based system. We used a small-size linear probe that can be located next to the chamber due to small-size of both, the probe and the cup. A practical applicability of the novel methodology is discussed later in this report.

### Level of Noise Produced by the Device During Its Operation

Similar to Pinheiro et al. (2020), relatively noiseless operation was achieved by employing a silent pump, negative or positive pressure tank and a release valve. The pump generates the negative/positive pressure in the tank immediately before the delivery of the pressure to the chambers and the pressure is delivered by opening the release valve. During the chambers pressurizing, the pump does not work and the system constitutes a fully enclosed space thereby requiring a perfect sealing (between the chambers and the neck) to avoid any loss of the negative/positive pressure developed at start. In order to ensure the perfect sealing, the pressurized space beneath the cup was enclosed by a thin membrane stuck to the skin.

The average noise generated by our device (∼35 dB) is similar to that of Pinheiro’s apparatus (∼34 dB) and substantially lower than the noise produced by other custom-made devices employing the parts of vacuum cleaners and/or compressors (∼75 dB) (Raine and Cable, 1999; Pinheiro et al., 2020).

### Effectiveness of the Device in Baroreceptor Stimulation/Unloading

The major strength of our approach is that the carotid artery diameter was monitored throughout the entire episode of NS or NP, thereby providing a direct measure of the intensity of baroreceptor stimulation.

Efficiency of the transmission of external pneumatic pressure applied over the subject’s neck to the carotid sinus has been questioned repeatedly (Ludbrook et al., 1977; Cooper and Hainsworth, 2009). Two studies measured the pressure in the jugular vein (adjacent to the carotid sinus) during the test and demonstrated that 100% (Eckberg, 1976) or 64% (Ludbrook et al., 1977) of negative pressure and 86% of positive pressure is transmitted. Along this line, Kober and Arndt (1970) reported linear relationship between the pressure inside the chamber (within the range of ± 45 mmHg) enclosing the subject’s head and neck and the diameter of carotid common artery as assessed with US.

The performance of the testing equipment is rarely verified by the methods mentioned above, however, and most authors rely on the pattern of physiological response to the NS/NP instead. Heart rate responses obtained with our device are qualitatively and quantitatively similar to those reported by other studies. Carotid–HR baroreflex function curve parameters were coherent with data shown previously for both paired neck chambers (Raine and Cable, 1999; Ogoh et al., 2002) and neck collars (Potts et al., 1993; Ogoh et al., 2002). Indeed, Ogoh et al. (2002) found these paired neck chamber results highly comparable to the “standard neck collar” ones. Consistently, we have also noted similarities between the magnitude of HR responses to the selected NP/NS values and those reported for neck collars, i.e., mean decrease of about 6 ± 2 (SEM) bpm for −30 mmHg NS (Ogoh et al., 2002) and 11 ± 3 bpm for −60 mmHg NS (Huang et al., 2016; Pinheiro et al., 2020); mean increase of about 5 ± 1 bpm for 30 mmHg NP (Ogoh et al., 2002); mean increase of about 8 ± 3 bpm for 40 mmHg NP (Huang et al., 2016).

Mean arterial pressure responses revealed higher variability and certain discrepancies compared to other studies. Although the saturation, response range and centering/operating point values we obtained corroborated those reported in the literature (Potts et al., 1993; Raine and Cable, 1999; Ogoh et al., 2002), we have noted relatively low threshold (mean value of about 22 mmHg, compared to >50 mmHg shown in other studies [Potts et al., 1993; Ogoh et al., 2002]). Additionally, MAP responses to the selected NP/NS values were lower than those reported by other authors (e.g., drop of about 7 ± 4 mmHg for −60 mmHg, vs. ∼15−∼17 mmHg [Huang et al., 2016; Pinheiro et al., 2020]). We speculate it might be due to the delay in sympathetic nerve activity and (consecutive) vascular resistance alterations (Ogoh et al., 2003b), as well as in the blood pressure data collection, making our stimulus duration too short for the proper evaluation of MAP response. Indeed, several works demonstrated that the stimulus timeframe of 5 s may not suffice to obtain the peak/nadir of the MAP response, as the blood pressure may rise/decline progressively throughout the first 10–15 s of NP/NS (Ogoh et al., 2003b; Huang et al., 2016).

### Carotid Artery Ultrasonography During the Neck Suction/Pressurizing

Numerous studies have used ultrasound carotid artery imaging during the pharmacological assessment of baroreflex function which is based on physiological responses to transient blood pressure changes induced by iv infusion of vasoactive drug(s) (Hunt et al., 2001; Taylor et al., 2014). This method, originally proposed by Hunt et al. (2001) allows for isolating the mechanical component from the neural component of the carotid baroreflex.

In contrast, ultrasound vascular imaging during the NS or NP has been rarely performed, perhaps due to technical difficulties. Most systems require the subject’s neck to be wrapped tightly by the chamber/collar, to ensure a good seal, and therefore the carotid artery is not accessible for ultrasound examination. To our knowledge, only two previous studies attempted to overcome this limitation. Ogoh et al. (2002) used simplified paired neck chamber adapted from ear protectors to enable pulse Doppler measurement of ascending aortic blood flow velocity during the application of variable-pressure to the subject’s neck. Using this setup, they found that the carotid baroreceptor stimulation or unloading does not affect stroke volume. In another study, Kober and Arndt (1970) utilized the box enclosing the subjects head and neck and found a linear relationship between the pressure in the box and the common carotid artery diameter as assessed with US. The results of these studies are not applicable to the contemporarily used neck chamber setups. In the study by Ogoh et al. (2002), the ascending aorta was examined with US, not the carotid artery. Although the aortic and carotid stiffness were shown to be correlated in healthy subjects, this relation was rather weak-to-moderate (*r* = 0.64) (Paini et al., 2006) and therefore changes in ascending aortic diameter cannot be considered a reliable marker of the changes in carotid artery diameter. Regarding the study by Kober and Arndt (1970), the potentially confounding effect of the altered blood flow in the subject’s head should be highlighted. In fact, changes in carotid artery diameter might result primarily from external pressure-induced changes in blood circulation in the head. Therefore, the question whether the negative or positive pressure applied to the subject’s neck, selectively to the small areas over the carotid sinuses can visibly affect the carotid sinus diameter remains unanswered. Furthermore, neither of these works provide an easily applicable tool allowing the carotid artery to be visualized with US during the application of negative or positive pressure to the subject’s neck.

In the current study, we have demonstrated that the variable-pressure applied on the subject’s neck via small cups acutely affects the carotid artery diameter, and it is feasible to track these changes with US imaging utilizing small-size linear probe. Furthermore, we found linear, dose-dependent relation between the external pressure applied on the subject’s neck and the systolic-diastolic amplitude of the carotid artery.

### Other Advantages and Disadvantages of the Novel Device

Our method allows for unilateral, left- or right-side stimulation/unloading of the carotid baroreceptors. The issue of functional baroreflex asymmetry (hysteresis) may be of clinical significance, given that right-side stimulation was shown to be more effective than the left-side or bilateral stimulation (De Leeuw et al., 2015). Furthermore, due to relatively small size, our system is fully portable and easy to set in each laboratory.

In the current version of the neck chamber set, the cups are mounted on a rigid frame firmly attached to the bed. This ensures stability of the whole set, thereby permitting an undisturbed US recording, but precludes the baroreflex examination during large muscle mass, two-limbs dynamic exercises in standing or sitting position (i.e., treadmill running or cycling on an ergometer). Static or dynamic handgrip or supine two-legs cycling could be used, however. Furthermore, we believe that minor redesigning of the frame would make the neck chamber set suitable for use during the exercise on cycloergometer, as similar experiments employing large and heavy neck collars were performed before (i.e., Ogoh et al., 2013). Recently, Paliwal et al. (2020) presented a clever strap- and frame-free solution for gripping the neck chamber over the subject’s neck with multiple small suction cups located on the outer brim of the actual positive and negative pressure chamber. Adapting Paliwal’s approach in our system would greatly facilitate the variable-pressure application during the exercise.

Pinheiro’s apparatus enables the NS delivery to be synchronized with the R-wave of ECG recording. The early version of our device, presented in the study did not allow for synchronizing the NS/NP with the ECG signal. Therefore, we did not measure the distance between the R-wave and the pressure delivery time. However, a minor modification of our device would enable an alternative approach utilizing The Event Manager add-on for LabChart software and digital output of the data acquisition system (PowerLab, ADInstruments) connected to the neck chamber device. That approach has been used in the previous experiment from our laboratory to synchronize the delivery of transcutaneous auricular electrostimulation with the inspiratory- or expiratory phase of the breathing cycle (Paleczny et al., 2019). In fact, virtually all biological signals recorded with the LabChart and Power Lab system can be used as a trigger for NS/NP delivery, according to user-defined criteria (i.e., rise or fall of a given parameter above/below pre-specified threshold). Furthermore, this approach would allow for synchronizing the NS/NP delivery with more than one signal at the same time (i.e., respiratory phase and ECG phase).

Our appliance is not capable of providing pulsatile wave of negative/positive pressure. However, Ogoh et al. (2003a) demonstrated that there is no difference in the physiological effects of sustained vs. pulsatile pressure.

## Conclusion

We have demonstrated that the novel custom-built device allows for acute, low-noise stimulation and unloading of the carotid baroreceptors, with negative and positive pressures, respectively, generated beneath the small, 3D-printed cups positioned on the subject’s neck over the carotid sinuses. Consistent and dose-dependent changes in HR during the NS and NP were observed in all study participants. Furthermore, we have shown that a reduced size and asymmetrical, one-side flattened shape of the cups enables positioning the US probe in close proximity to the area of NS and NP. US imaging of the carotid artery during the application of the negative and positive pressure is feasible with our device and allows for direct tracking of the carotid distension (during NS) or compression (during NP). We have found linear, dose-dependent relation between the pressure applied on the subject’s neck and the change in the systolic-diastolic difference in the carotid artery diameter as assessed with US.

## Data Availability Statement

The original contributions presented in the study are included in the article/Supplementary Material, further inquiries can be directed to the corresponding author.

## Ethics Statement

The studies involving human participants were reviewed and approved by Bioethics Committee of the Wrocław Medical University. The patients/participants provided their written informed consent to participate in this study.

## Author Contributions

RS and BPa conceived and designed the experiment. PM designed and constructed neck chamber device. RS, TO, ST, and BPa performed the experiments, analyzed the data, and interpreted the results. RS, TO, PM, and BPa drafted and edited the manuscript. All authors critically revised the manuscript and approved the final version of the manuscript.

## Conflict of Interest

PM was employed by IMER Systems, Wrocław, Poland. The remaining authors declare that the research was conducted in the absence of any commercial or financial relationships that could be construed as a potential conflict of interest.

## Publisher’s Note

All claims expressed in this article are solely those of the authors and do not necessarily represent those of their affiliated organizations, or those of the publisher, the editors and the reviewers. Any product that may be evaluated in this article, or claim that may be made by its manufacturer, is not guaranteed or endorsed by the publisher.
