# Supplementary material for: Neck Chamber Technique Revisited: Low-Noise Device Delivering Negative and Positive Pressure and Enabling Concomitant Carotid Artery Imaging With Ultrasonography
Source: Front Physiol. 2021 Oct 5;12:703692. doi: 10.3389/fphys.2021.703692 (PMC8525882; doi:10.3389/fphys.2021.703692)

Supplementary file

Figure S-1. A graph illustrating the negative (panel A) and positive (panel B) pressures generated beneath the neck chambers. Mean±standard deviation of the variable-pressure profile calculated from all experiments performed was shown as a blue line with shade. The variable-pressure profile obtained with the chambers applied on a rigid, glass surface was shown as a red line. The values are mean differences between the onset of pressure application and the pressure at 5th second of the pressure application.

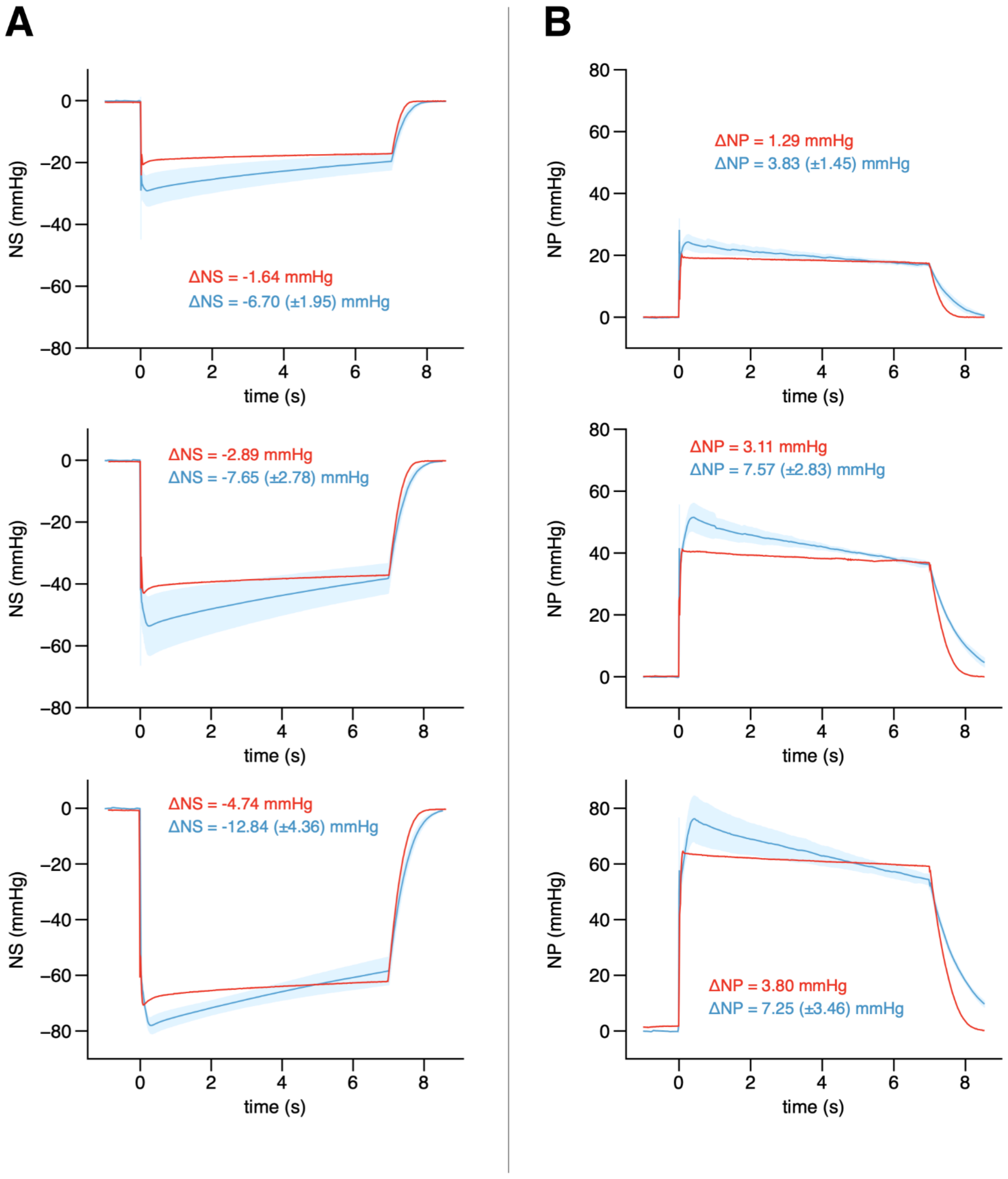

Supplement: Supplementary file 1 [file Data_Sheet_1.PDF]
